# Upper thoracic spinal schwannoma leading to intracranial hypertension and hydrocephalus: A case report and literature review

**DOI:** 10.1097/MD.0000000000041889

**Published:** 2025-04-25

**Authors:** Lianjie Li, Yongping Zhang, Dongxiao Pan, Lan Cheng

**Affiliations:** a Department of Neurosurgery, Ningbo No. 2 Hospital, Ningbo, Zhejiang, China; b Department of Histopathology, Ningbo Clinicopathological Diagnosis Center, Ningbo, Zhejiang, China; c Department of Cardiovascular Medicine, Ningbo No. 2 Hospital, Ningbo, Zhejiang, China.

**Keywords:** case report, hydrocephalus, intracranial hypertension, schwannoma, upper thoracic spine

## Abstract

**Rationale:**

Hydrocephalus is predominantly caused by intracranial hemorrhage, infections, tumors, genetic metabolic disorders, and congenital malformations. Hydrocephalus secondary to spinal cord tumors is exceedingly rare; these tumors are predominantly located in the upper cervical spinal region or near the conus medullaris. Hydrocephalus and elevated intracranial pressure caused by upper thoracic spinal schwannomas have not previously been reported.

**Patient concerns:**

We report the first case in a 71-year-old female patient who presented with bilateral lower limb weakness for 6 months, accompanied by headache, dizziness, and urinary incontinence for 1 month.

**Diagnoses:**

This patient was diagnosed with schwannomas located in the upper cervical spinal region.

**Interventions:**

Based on preoperative examination results, it was difficult to distinguish a schwannoma from a spinal meningioma. Subsequently, a microsurgical operation was performed, and the whole tumor was removed via a posterior approach. Tumor tissue histopathological results revealed a whole capsule; under a light microscope, the tumor mainly consisted of sheath cells, which were arranged in a palisade or swirl shape. Antoni A and Antoni B regions constituted a large part of the whole tumor. Moreover, cytologic atypia and necrosis or mitosis were absent. Immunohistochemical staining revealed strong positivity staining for S-100 and SOX 10. Hence, a histopathological diagnosis of schwannomas was finally confirmed. The patient’s symptoms of intracranial hypertension, hydrocephalus, and spinal cord dysfunction were significantly alleviated after the operation.

**Outcomes:**

Follow-up magnetic resonance image (1 month after being discharged from the hospital) of the thoracic spine and brain revealed complete resection of the upper thoracic spinal schwannoma, a significant reduction in the size of ventricles, and marked alleviation of periventricular interstitial edema.

**Lessons:**

The findings emphasize the importance of considering spinal tumors in cases of unexplained hydrocephalus and may guide early microsurgical intervention.

## 1. Introduction

Hydrocephalus is a rare complication of spinal tumors, accounting for only 6.3% of such cases.^[1]^ Hydrocephalus with intracranial hypertension caused by the upper thoracic spinal schwannoma has not been reported yet, and the pathogenesis is complex. Particularly in patients exhibiting no significant spinal cord dysfunction, it is easily misdiagnosed and often leads to inappropriate shunt placement during treatment.^[2]^ Herein, we reported a rare case with intracranial hypertension and hydrocephalus caused by a spinal schwannoma located in the upper thoracic, a particular site that had never been reported yet. Furthermore, we also performed a systematic review of the literature reported previously on spinal schwannoma with intracranial hypertension and hydrocephalus for clinical and pathological features.

## 2. Case presentation

### 2.1. Clinical characteristics

**Figure 1.**
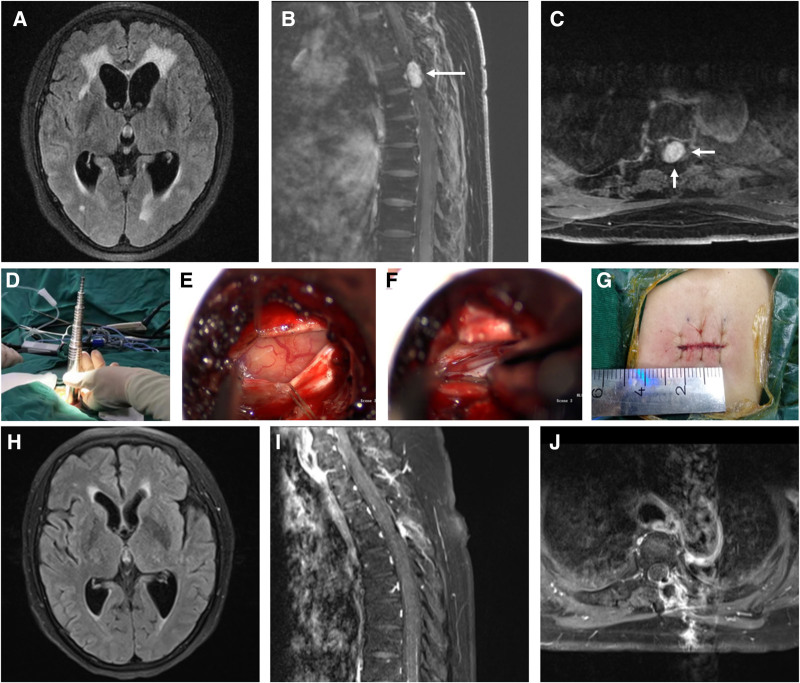
Preoperative, intraoperative, and final follow-up magnetic resonance image in the brain and spine. Preoperative horizontal plane revealed hydrocephalus, axial view of the T2 FLAIR MRI of the head showed ventricular enlargement and periventricular interstitial edema (A). The sagittal and horizontal plane of enhanced magnetic resonance images revealed an ellipsoid mass with well-defined demarcation in the upper thoracic spinal canal (B, C). Intraoperatively established channel system through a 2-cm surgical incision, then the dura mater was incised to reveal the tumor during the surgery (D, E). Intraoperative image showed the complete resection of the tumor (F). A 2-cm surgical incision lateral to the midline at the T4 level is shown (G). At the final follow-up, the MRI scan of the head revealed no recurrence of the tumor and signs of hydrocephalus relieved (H–J). FLAIR, fluid-attenuated inversion recovery; MRI, magnetic resonance imaging.

This study was approved by the Ethics Committee of Ningbo No. 2 Hospital. Written informed consent was obtained from the patient and his family members for the publication of this case report and the accompanying images. This case report was exempt from institutional review board review. A 71-year-old female patient presented with complaints of bilateral lower limb weakness for 6 months, accompanied by headache, dizziness, and urinary incontinence for 1 month. The patient had no history of intracranial hemorrhage, infection, tumors, genetic metabolic disorders, or congenital malformations. Physical examination revealed that the patient was conscious, with bilateral papilledema and visual acuity of 0.6 in both eyes. Significant declines in memory, calculation, and cognitive abilities were observed. Muscle strength and tone in the upper limbs were normal, while the right lower limb had grade III muscle strength and the left lower limb had grade II muscle strength, with normal muscle tone in both lower limbs. Touch, pain, and temperature sensation were intact in all 4 limbs. Abdominal reflex was normal, with no signs of nuchal rigidity or pathological reflexes. Upon admission, the plain magnetic resonance imaging (MRI) scan of the head revealed patchy abnormal signals in the periventricular white matter around the anterior and posterior horns and the body of the lateral ventricles. These signals appeared hypointense on T1-weighted imaging, hyperintense on T2-weighted imaging, and hyperintense on fluid-attenuated inversion recovery images, suggesting interstitial edema and ventricular dilation (Fig. 1A). The plain and contrast-enhanced MRI scans of the thoracic spine revealed a nodular abnormal signal on the left side within the spinal canal at the T4 level, measuring approximately 2.0 cm × 1.2 cm × 1.0 cm. The lesion exhibited hypointensity on T1-weighted imaging and hyperintensity on T2-weighted imaging, with significant enhancement postcontrast (Fig. 1B, C). A lumbar puncture was performed, revealing a cerebrospinal fluid (CSF) pressure of 265 mmH_2_O (1 mmH_2_O = 0.00978 kPa). The CSF appeared clear and transparent, with a white blood cell count of 5 × 10^6^/L, glucose concentration of 3.67 mmol/L, protein level of 1.03 g/L, and chloride concentration of 121.4 mmol/L. These findings led us to suspect the diagnosis was probably a schwannoma or a spinal meningioma.

Subsequently, the patient underwent microsurgical resection of the tumor at the T4 level via a posterior approach. For the surgical procedure, the prone position was used. With the preoperative localization point (between the T4 and T5 vertebrae) as the center, a 2-cm longitudinal incision was made adjacent to the midline of the back, cutting through the skin, fascia, and muscle layer by layer. A guide pin was inserted at the T4/T5 laminar junction, followed by the insertion of the dilator to retract the muscle and the placement of a tubular retractor with the appropriate size. Under a microscope, the spinous process and articular processes of the T4 vertebra were identified, followed by partial removal of the spinous process and lamina of the T4 vertebra using a drill. After achieving complete hemostasis, a longitudinal incision was made in the dura mater, which was then retracted and suspended with sutures to expose the lesion. Exploration revealed that the tumor was located dorsally and slightly to the left of the spinal cord, with tight adhesions to the spinal cord and nerve roots. Upon retracting the spinal cord and nerve roots, the tumor was found to be relatively large. Consequently, the tumor was gradually resected in sections, with repeated dissection and removal, continually separating adhesions between the tumor and the spinal cord and nerve roots, ultimately achieving complete excision of the lesion (Fig. 1D–G). After confirming the absence of bleeding through irrigation, the dura mater was meticulously repaired and sutured, followed by layer-by-layer closure of the muscle, fascia, subcutaneous tissue, and skin. One month after surgery, a repeat plain MRI scan of the head indicated a reduction in the patchy abnormal signals in the periventricular white matter around the anterior and posterior horns and the body of both lateral ventricles. Additionally, the size of the ventricles decreased significantly compared with preoperative measurements (Fig. 1H). The plain and contrast-enhanced MRI scans of the thoracic spine indicated complete resection of the tumor (Fig. 1I, J).

### 2.2. Histopathological findings

#### 2.2.1. Microscopic features

**Figure 2.**
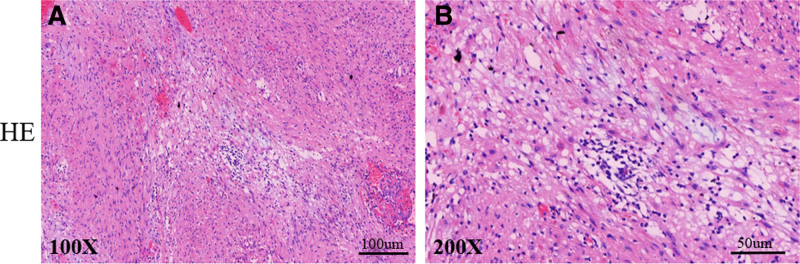
HE (hematoxylin–eosin) staining. The tumor consists of a fascicular area (Antoni A) and a reticular area (Antoni B), shown in (A) (×100) and (B) (×200).

A clear radius and whole capsule were observed in the operation. The whole lump was resected and tissue slides were made for HE (hematoxylin–eosin) and immunohistochemical staining (Fig. 2A, B). The mucous structure and blood vessels were identified. The tumor mainly consisted of a fascicular area (Antoni A) and a reticular area (Antoni B), with migration observed in both zones.

#### 2.2.2. Immunohistochemical staining

**Figure 3.**
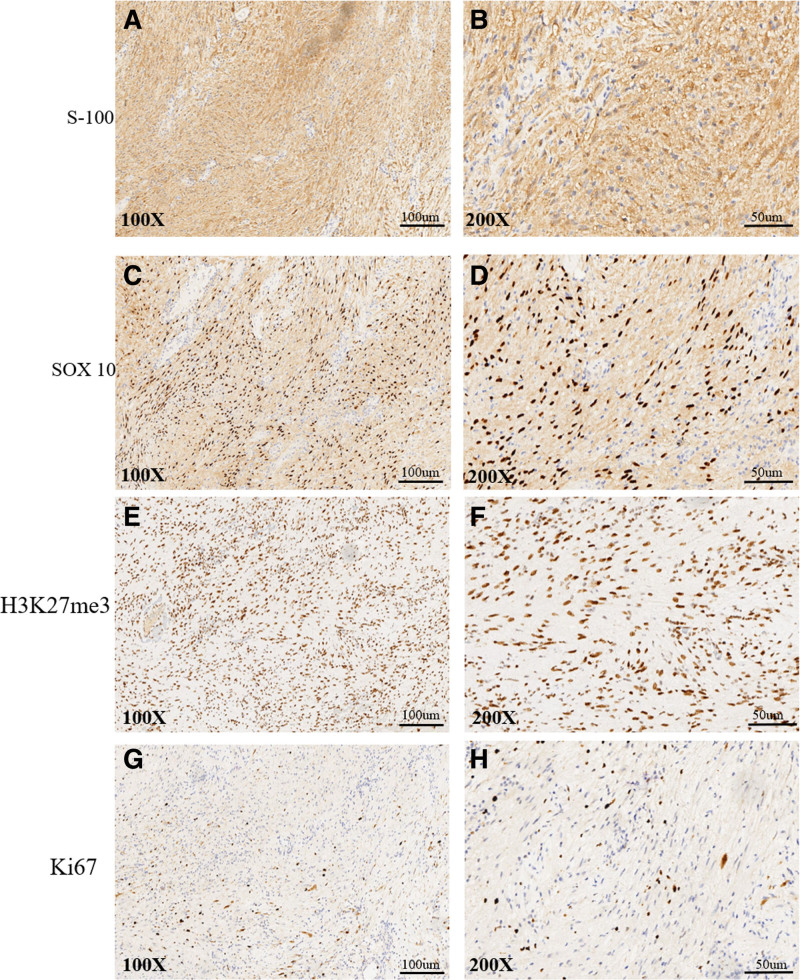
Immunohistochemical staining. S-100 was positively stained in the cytoplasm and the nucleus of tumor cells, shown in (A) (×100) and (B) (×200). Tumor cells showed nuclear positivity for SOX 10 (C, D). (E, F) Strong positivity for H3K27me3 was detected in the nucleus. About 2 to 5% of nucleus was positively stained with Ki67, shown in (G, H).

Immunohistochemical staining showed a strong and diffuse positive signal, mainly localized in the cytoplasm and nucleus for S-100 (Fig. 3A, B). A strong positive signal was also observed in the nucleus for SOX10 (Fig. 3C, D). To distinguish samples from malignant tumors, we conducted H3K27me3 and Ki67 immunohistochemical staining; a strong positively stained nucleus was observed in H3K27me3 (Fig. 3E, F), and below 5% positive cells were stained with Ki67 (Fig. 3G, H).

## 3. Discussion

Hydrocephalus by spinal tumors can be classified into increased pressure and normal pressure hydrocephalus.^[3,4]^ It is more commonly associated with highly malignant intramedullary astrocytomas and ependymomas.^[5]^ Spinal tumors frequently occur in the upper cervical segment or near the conus medullaris. Kushel’ et al^[2]^ reported that, among 541 cases of spinal tumors, 32 were complicated with hydrocephalus, 19 of which (59.4%) were located at the upper cervical cord. In 1931, Kyrieleis reported the first case of a conus medullaris tumor presenting with increased intracranial pressure. Subsequent cases of hydrocephalus resulting from conus medullaris tumors have been documented. Hydrocephalus with intracranial hypertension caused by an extramedullary intradural schwannoma in the upper thoracic spine is exceedingly rare.

In this case, the patient was admitted with symptoms of spinal cord dysfunction and hydrocephalus. CSF pressure was elevated, and the patient had no history of intracranial hemorrhage, infection, tumors, genetic metabolic disorders, or congenital malformations. Following tumor resection through transcranial microsurgery, the patient’s symptoms of intracranial hypertension and hydrocephalus improved significantly. In this case, the pathological findings point to schwannoma properties. Schwannomas, also known as neurilemmomas, neuromas, or neurinomas, originate from Schwann cells of the peripheral nerve sheath.^[6]^ Schwannomas, neurofibromas, and malignant peripheral nerve sheath tumors are derived from nerve sheath.^[7]^ Typical schwannomas often present as an encapsulated lump. They consist of 2 different morphological features, shown as Antoni A and Antoni B areas in different ratios.^[8]^ S-100 protein, a specific marker, is prevalent in Antoni A areas and is used to confirm the diagnosis of schwannoma and benign nerve sheath tumors.^[8,9,10]^ In the present case, strong positive signals for S-100 and SOX10 were observed in the immunohistochemical staining.

Moreover, staining for the proliferation marker Ki67 was also performed in this case, which indicated a low proliferation index (<5%). Deletion of H3K27me3 is often used as an adjunct detection marker for malignant peripheral schwannomas and we displayed strong positively stained nuclei in H3K27me3 immunohistochemical staining in the present case.

Previously, cases of intracranial hypertension and hydrocephalus caused by spinal tumors have been reported. Herein, we reviewed 9 cases previously reported in the literature, and their clinical features are shown in Table 1. Two cases presented as hydrocephalus located at L2 and cauda, both of which were confirmed schwannoma.^[11,13]^ One case displayed papilledema only and was pathologically diagnosed as ependymoma.^[11]^ Six cases showed intracranial hypertension and hydrocephalus, mainly located at the cervical segment, inferior thoracic segment, lumbar segment, and cauda.^[11,12,14,15]^ It has been reported that oligodendroglioma often grows slowly in the white matter of the brain and contributes to intracranial pressure, HE staining showed cell swelling, clear light, and obvious nuclear atypia.^[16]^

**Table 1:** Clinical features of 9 cases.

Case no.	Age (yr)/sex	Site	Tumor type	Presentation	Papilledema	Hydrocephalus	References
1	49/Female	T12	Neurofibroma	Headache, memory loss, gait disturbance	+	+	Ridsdale and Moseley^[11]^
2	62/Female	L2	Schwannoma	Gait disturbance, memory loss, confusion	-	+	Ridsdale and Moseley^[11]^
3	44/Female	T11	Ependymoma	Back and leg pain	+	-	Ridsdale and Moseley^[11]^
4	32/Male	T12	Oligodendroglioma	Headache, visual disturbance	+	+	Ridsdale and Moseley^[11]^
5	21/Male	L5	Oligodendroglioma	Headaches, confusion, visual disturbance	+	+	Ridsdale and Moseley^[11]^
6	53/Female	L3/L4	Schwannoma	Headache, blurry vision, communicating hydrocephalus, and intracranial hypertension	+	+	Ishaque et al^[12]^
7	59/Female	Cauda	Neurinoma	Disorientation, gait disturbance, impaired superficial sensation	-	+	Niwa et al^[13]^
8	58/Male	C3/4	Schwannoma	Ataxic broad-based gait, confusion, disorientation, mild lethargy	+	+	Bland and McDonald^[14]^
9	63/Male	Cauda	Neurinoma	Visual disturbance	+	+	Zhou et al^[15]^

In the imaging studies, we confirmed a reduction in periventricular interstitial edema and a significant decrease in the size of the ventricles. Therefore, hydrocephalus in this patient was caused by a schwannoma located in the upper thoracic segment. Moreover, at the 1-year postoperative follow-up, the patient exhibited improvements in headaches, dizziness, and urinary incontinence, along with significant enhancements in memory, calculation, and cognitive abilities. The muscle strength in the right lower limb was assessed at grade V, and that of the left lower limb at grade IV.

The precise mechanism by which upper thoracic spinal tumors contribute to intracranial hypertension and hydrocephalus remains unclear. It is currently believed that intracranial hypertension associated with spinal tumors is related to 2 main factors, alterations in CSF dynamics and alterations in CSF composition. Alterations in CSF dynamics. On the one hand, CSF flows from the cranial cavity into the spinal subarachnoid space, where it is absorbed through the spaces around blood vessels and nerve roots. Spinal tumors can directly compress the subarachnoid space, causing mechanical obstruction of the CSF circulation pathway. This obstruction impairs CSF absorption below the level of obstruction, thereby leading to intracranial hypertension. On the other hand, spinal tumors can compress the venous plexus within the spinal canal and the veins on the surface of the spinal cord, leading to changes in venous pressure and osmotic pressure. This disruption impairs CSF absorption and ultimately results in intracranial hypertension.^[17]^ Additionally, animal studies have identified the lateral aspect of the conus medullaris as a primary pathway for CSF outflow, playing a crucial role in the circulation and absorption of CSF. Tumors located near the conus medullaris can easily obstruct this outflow pathway, leading to intracranial hypertension.^[17]^ Alterations in CSF composition, which are primarily related to the entry of tumor exudates, secretions, and cells following hemorrhage and rupture into the CSF. In most patients with spinal tumors, protein levels in CSF are elevated, which can increase the viscosity of the CSF, leading to subarachnoid adhesions. This impedes the passage of CSF through the semipermeable membrane, thereby affecting CSF absorption and subsequently resulting in intracranial hypertension. The etiology of spinal tumors complicated with intracranial hypertension is complex, involving multiple factors. Based on this case study, we hypothesize that the causes of intracranial hypertension may include the compression of the subarachnoid space, the venous plexus within the spinal canal, and the veins on the surface of the spinal cord by an upper thoracic spinal schwannoma, as well as elevated protein levels in CSF.

The treatment of upper thoracic spinal tumors complicated with intracranial hypertension and hydrocephalus should be guided by the specific location and characteristics of the lesion, employing tailored surgical strategies. In patients with spinal tumors complicated with hydrocephalus, preoperative shunt placement should be avoided for 2 primary reasons: hydrocephalus often resolves following the resection of the spinal lesion, particularly when the lesion is extramedullary, and preoperative shunt placement may lead to shunt-related neurological deterioration.^[2]^ For intramedullary gliomas, a contrast-enhanced MRI scan should be conducted to exclude the possibility of hydrocephalus caused by intracranial leptomeningeal metastasis. In such cases, the likelihood of hydrocephalus resolving after tumor resection is low. If intracranial hypertension persists, shunt surgery should be performed concurrently with spinal tumor resection or immediately afterward. In addition, if hydrocephalus develops late after the resection of a spinal tumor and the primary lesion is an intramedullary glioma, hydrocephalus should be considered an early indicator of intracranial tumor metastasis. Performing shunt surgery in cases where a malignant spinal tumor is undiagnosed and unresected, incompletely resected, or locally recurrent can lead to CSF reflux and leptomeningeal dissemination of the tumor.^[18]^ Elevated CSF protein levels contribute to the development of hydrocephalus. Researchers have linked elevated CSF protein levels and the resultant rise in CSF viscosity to intracranial hypertension. Following the surgical resection of benign spinal tumors, compression of the spinal cord is relieved, CSF circulation is restored, and postoperative CSF protein levels decrease, typically resulting in the alleviation of hydrocephalus symptoms.^[19,20]^ The patient presented with an upper thoracic spinal schwannoma, classified as a benign extramedullary tumor. Surgical resection is the primary approach to relieve spinal cord compression and alleviate symptoms related to hydrocephalus and intracranial hypertension. In terms of surgical approach selection, traditional open laminectomy has significant drawbacks, such as substantial intraoperative blood loss, a high incidence of postoperative complications, pronounced postoperative pain, and slow recovery.^[21]^ In recent years, surgical procedures have evolved toward minimally invasive techniques, particularly in spinal and spinal cord surgeries. Compared with traditional open surgery, minimally invasive approaches reduce the incidence of postoperative adverse events significantly, such as CSF leaks, infections, and postoperative bleeding, while also improving patient experiences related to pain and muscle injury.^[22]^ Microsurgical resection of the tumor via a microchannel approach minimizes patient trauma, accelerates recovery, and yields satisfactory outcomes.

In summary, for cases of unexplained hydrocephalus, the possibility of a spinal tumor should be considered. The pathogenesis of upper thoracic spinal tumors complicated with intracranial hypertension and hydrocephalus is complex and warrants further investigation. Surgical resection of the tumor is crucial for treatment and caution should be exercised against premature shunt placement.

## Acknowledgments

We wish to thank Prof Shouseng Wang of The 900th Hospital of the Chinese People’s Liberation Army Joint Logistic Support Force for the guidance of this manuscript. We would also like to thank Editage (www.editage.cn) for English language editing.

## Author contributions

**Supervision:** Lan Cheng.

**Writing – review & editing:** Lan Cheng.

**Writing – original draft:** Lianjie Li.

**Investigation:** Yongping Zhang.

**Data curation:** Dongxiao Pan.
